# The prognostic value of galactosylceramide-sulfotransferase (Gal3ST1) in human renal cell carcinoma

**DOI:** 10.1038/s41598-021-90381-6

**Published:** 2021-05-25

**Authors:** Stefan Porubsky, Malin Nientiedt, Maximilian C. Kriegmair, Jörn-Helge Heinrich Siemoneit, Roger Sandhoff, Richard Jennemann, Hendrik Borgmann, Timo Gaiser, Cleo-Aron Weis, Philipp Erben, Thomas Hielscher, Zoran V. Popovic

**Affiliations:** 1 Institute of Pathology, University Medical Center Mannheim, University of Heidelberg, Theodor-Kutzer-Ufer 1-3, 68167 Mannheim, Germany; 2 Department of Urology and Urosurgery, University Medical Center Mannheim, University of Heidelberg, Mannheim, Germany; 3 Lipid Pathobiochemistry Group, German Cancer Research Center, Heidelberg, Germany; 4 Department of Urology, University Medical Center, Johannes-Gutenberg University, Mainz, Germany; 5 Department of Biostatistics, German Cancer Research Center, Heidelberg, Germany; 6 Institute of Pathology, University Medical Center, Johannes-Gutenberg University, Mainz, Germany

**Keywords:** Oncology, Pathogenesis, Risk factors, Urology, Tumour biomarkers, Urological cancer, Renal cancer

## Abstract

Renal cell carcinoma (RCC) is the deadliest primary genitourinary malignancy typically associated with asymptomatic initial presentation and poorly predictable survival. Next to established risk factors, tumor microenvironment may alter metastatic capacity and immune landscape. Due to their high concentrations, sulfoglycolipids (sulfatides) were among the first well-described antigens in RCC that are associated with worse prognosis. As sulfatide detection in routine diagnostics is not possible, we aimed to test the prognostic value of its protein counterpart, sulfatide-producing enzyme Gal3ST1. We performed retrospective long-term follow up analysis of Gal3ST1 expression as prognostic risk factor in a representative RCC patient cohort. We observed differentially regulated Gal3ST1 expression in all RCC types, being significantly more associated with clear cell RCC than to chromophobe RCC (p = 0.001). Surprisingly, in contrast to published observations from in vitro models, we could not confirm an association between Gal3ST1 expression and a malignant clinical behaviour of the RCC. In our cohort, Gal3ST1 did not significantly influence progression-free survival (Hazard Ratio (HR): 1.7 95% CI (0.6–4.9), p = 0.327). Particularly after adjusting for histology, T-stage, N-status and M-status at baseline, we observed no independent prognostic effect (HR = 1.0 95% CI (0.3–3.3), p = 0.96). The analysis of Gal3ST1 mRNA expression in a TCGA dataset supported the results of our cohort. Thus, Gal3ST1 might help to differentiate between chromophobe RCC and other frequent RCC entities but—despite previously published data from cell culture models—does not qualify as a prognostic marker for RCC. Further investigation of regulatory mechanisms of sulfatide metabolism in human RCC microenvironment is necessary to understand the role of this quantitatively prominent glycosphingolipid in RCC progression.

## Introduction

Renal cell cancer (RCC), the most common neoplastic disease of the kidney and the most lethal urologic cancer, accounts for 5% and 3% for all cancers in men and women, respectively, and for approximately 2% of cancer-associated deaths worldwide^1–4^ with increasing disease burden in West Europe and North America^3^. Clear cell renal cell carcinoma (ccRCC), as the most frequent histological form, accounts for 80% of global RCC cases^5^. Other relatively frequent histological types include papillary RCC (pRCC—with type 2 typically presenting at higher stage and thus showing higher malignant potential than type 1) and usually less aggressive chromophobe RCC (chRCC). Rare histological types of RCC, including new entities introduced in the current WHO-classification, make < 5% of the total RCC pool. Although solely 10% of patients with localized renal malignant neoplasia show the ‘classical’ clinical trilogy (palpable mass, flank pain and hematuria), advanced clinical screening methods in the past years have led to an increased rate of early diagnosis and hence improved curability by radical surgery. Still, current data show that approximately 15% of RCC patients have metastases at initial presentation^1^ and that about one third of the patients with RCC will show metastatic progression after curative surgery^6^. The five-parameter multifactorial algorithm of the Memorial Sloan Kettering Center classification (MKSC) in addition to the International Metastatic Renal Cell Carcinoma Database Consortium (IMDC) are currently broadly used clinical prognostic models worldwide for metastatic RCC^7–10^. Although no specific prognostic biomarkers of the RCC microenvironment have been confirmed so far, recent transcriptomic studies have identified microenvironment-associated immune components that may be associated with RCC progression^11,12^. It is reasonable to assume that, like in other malignancies, dynamic changes of cellular, biophysical and biochemical factors of the RCC micromilieu may influence cancer progression^13^. The continuing search for clinical, biochemical, immunological and histological markers of RCC has led to a better understanding of tumor biology and disease progression, albeit with limited prognostic values^14^.

Glycosphingolipids are well established antigens recognized by unconventional T cells^15,16^. Sulfatides as a prominent group of polar glycosphingolipids represent antigens for human invariant natural killer T cells^17,18^. Enhanced expression of glycolipids in human RCC has been well documented decades ago^19,20^. Sulfated glycosphingolipids with their major representatives galactosylsulfatide (SM4s) and lactosylsulfatide (SM3) are detected in renal tissue under physiologic conditions where they may play important regulatory roles, for instance in adaptation to metabolic acidosis^21^. Increased expression of sulfatides in human renal cell cancer, but also in other cancer tissue and cell lines like adenocarcinoma of colon, lung, stomach and ovary^20,22–26^ may mediate metastatic spreading via binding to P-selectin^27^ and facilitate lymph node metastasis^28^. We have previously reported a sulfatide-mediated reprograming of macrophage expression profile towards a ‘M2’ (anti-inflammatory) phenotype via enhancement of apoptotic cancer cell uptake by sulfatide-coated macrophages in a murine experimental model^29^. Moreover, we have shown that sulfatides (including complex forms SM2 and SB1a) bind to chemokines^30^, together suggesting a complex role of sulfatides in shaping the immune landscape of RCC.

Two enzymes are crucial for the synthesis of sulfatides: in the first step, UDP-galactose ceramide galactosyltransferase (CGT) mediates synthesis of the sulfatide precursor galactosylceramide (GalCer) in endoplasmatic reticulum; in the second, upon transport of GalCer to Golgi apparatus, its sulfation occurs through cerebrosulfotransferase (CST; Gal3ST1)^31–34^. A recent in vitro study addressed potential pathways of Gal3ST1 gene overexpression in RCC and identified Gal3ST1 as a novel hypoxia-inducible factor (HIF) -mediated gene^35^. Furthermore, employing cell culture of different human kidney- and kidney cancer-lines, the authors suggest a potential role of Gal3ST1 in promotion of cancer immune escape via Gal3ST1-sulfatide-mediated cancer cell-platelet interaction and thereby worse prognosis^35^. Supporting this notion, the activation of platelets via binding of sulfatide to P-Selectin has previously been reported by other authors as well^36^.

The aim of our study was to evaluate the prognostic value of the sulfatide-producing enzyme Gal3ST1 expression in human RCC. Based on the current literature data, we hypothesized that overexpression of Gal3ST1 detected by immunohistochemistry in paraffin sections from patients with RCC reflects the malignant potential of RCC and negatively correlates with progression-free survival (PFS). In representative study cohort, our results showed no significant correlation between the intensity of Gal3ST1 staining and cancer progression, thereby opposing published data from cell culture models and denying Gal3ST1 as an independent histological prognostic marker in the clinical setting. In addition, our analysis of TCGA dataset from patients with ccRCC, pRCC and chRCC supported the results from our patient cohort, showing no significant prognostic relevance of Gal3ST1 mRNA expression. Hence, our results indicate that potential prognostic value of (lipid) sulfatide, as an abundant and in part immunomodulating component of RCC microenvironment, cannot be translated to (enzyme / protein) Gal3ST1 levels in tumor cells.

## Results

### Study cohort

Out of 119 patients with RCC included in the cohort, 94 had ccRCC (79%), 17 pRCC (14%, including 10 pRCC Type 1 and 7 pRCC Type 2 cases) and 8 chRCC (7%). Median age was 64 years (34–91 years) and 71% were male (n = 85). The median follow-up was 74 Months (IQR 57–83 Months) during which 29 events occurred. 53% of tumors were resected in pT1 stage (n = 63). Lymph node metastases were present in 4% (n = 5), while 10% (n = 12) patients had distant metastases at the time of surgery. Late metastases occurred in 19% (n = 24) cases, all of them with invasion of the vein wall described in the primary histopathologic diagnosis. Clinicopathological features of the study cohort are presented in the Table 1.

**Table 1:** Clinicopathological features of the study cohort.

Total number of patients	n = 119
Follow up* (months; range)	75 (0–105)
Sex (male, n; %)	85 (71.4)
Age* (years; range)	64 (34–91)
**Histological type (n; %)**	
ccRCC	94 (79)
pRCC	17 (14.3)
chRCC	8 (6.7)
**T stage (n; %)**	
pT1	63 (52.9)
pT2	18 (15.1)
pT3	36 (30.3)
pT4	2 (1.7)
Grade** (n; %)	
G1	14 (12.6)
G2	86 (77.5)
G3	11 (9.9)
N + (n; %)	5 (4.2)
M + (at the time of surgery, n; %)	12 (10.1)
M + (late, n; %)	24 (20.5)

*Data are presented as median value (with range in brackets).

**According to the current WHO classification, chRCC cannot be graded due to its innate nuclear atypia. Thereby the grading values shown here relate to ccRCC and pRCC cases.

### Increased expression of the enzyme Gal3ST1 can be detected in all major types of renal cell cancer but is less common in chRCC

**Figure 1:**
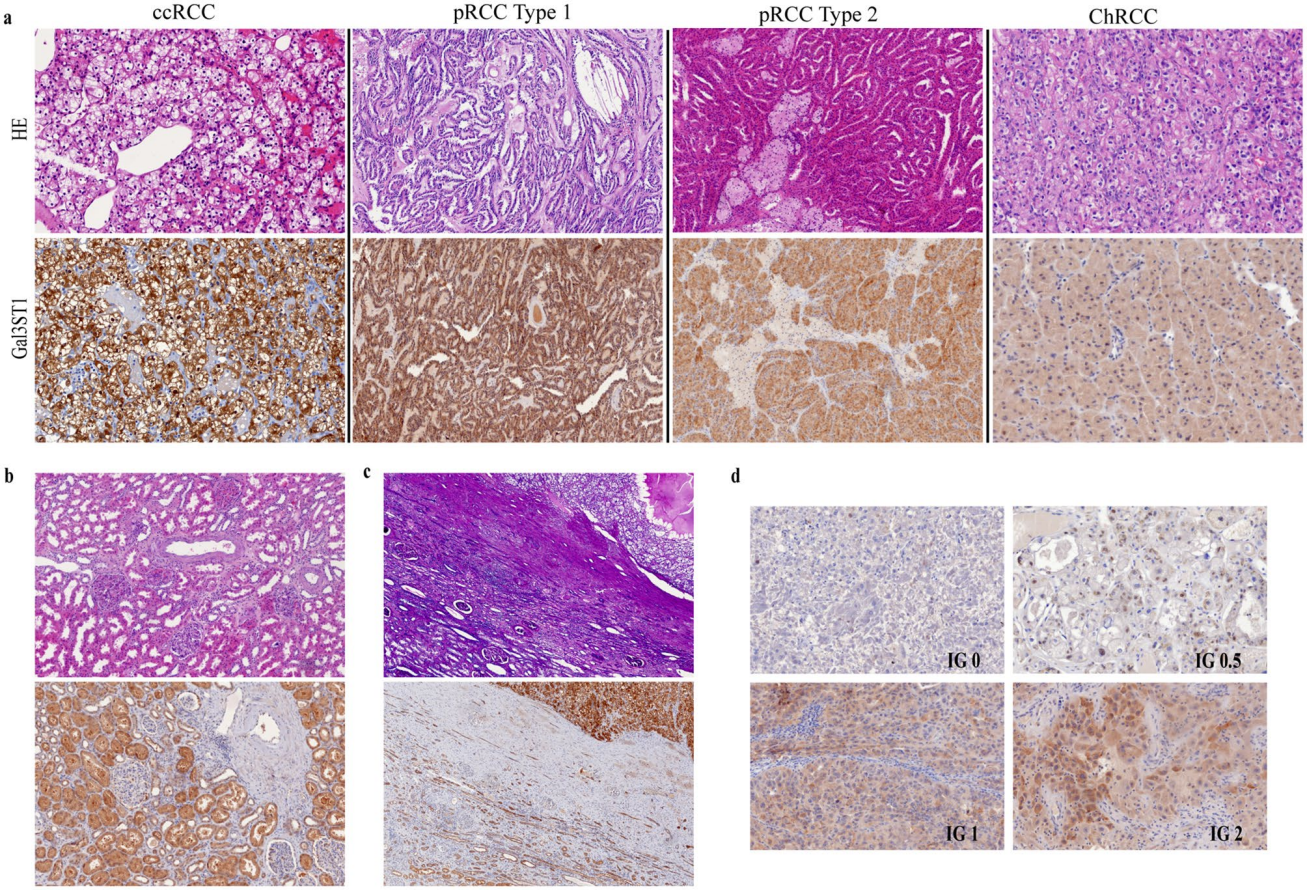
Immunohistochemical expression of Gal3ST1 in renal cell carcinoma. (**a**) cytoplasmatic and partially membranous positivity for Gal3ST1 could be observed in all common types of RCC. In this figure, Gal3ST1 expression in ccRCC and pRCC (type 1 and 2) was evaluated as highest intensity grade (IG = 3) and in shown ChRCC as IG = 1. (**b**) tumor-free kidney tissue typically shows physiological Gal3ST1 positivity of (proximal > distal) tubuli and negativity of glomeruli, interstitium and blood vessels. (**c**) peritumoral fibrotic tissue (mid part of the photomicrograph) is typically Gal3ST1 negative, while tumor (upper right corner) and peritumoral atrophic renal tubuli (lower left part) show strong or weak to moderate expression of Gal3ST1, respectively. (**d**) other patterns of Gal3ST1 distribution and intensity in RCC (IG—intensity grade). (**a**–**c**) HE staining – upper row, Gal3ST1 immunohistochemistry—lower row; (**a**,**d**): 200×, (**b**,**d**): 100 × magnification.

Paraffin sections from patients with renal malignancy (ccRCC, pRCC, chRCC) were analyzed for the localization and intensity of GAL3ST1-Expression in tumor and its microenvironment, including peritumoral kidney tissue. Specific, mainly cytoplasmic tumor cell positivity for Gal3ST1 could be detected in investigated histological cancer types, in various intensity grades (Fig. 1a,d). Tumor-free kidney (Fig. 1b) and peritumoral kidney tissue (Fig. 1c) showed typically weak to moderate Gal3ST1 expression, predominantly in tubular system (proximal > distal tubuli). Glomerular structures, blood vessels and interstitial inflammatory cells showed no specific Gal3ST1 staining, thereby serving as an internal negative control. The semiquantitative evaluation of the intensity of expression (intensity grade—IG) was performed as described below (Fig. 1d). In general, any degree of tumor-specific Gal3ST1 positivity was evident in 81% (n = 96) cases. Out of 94 ccRCC, 86% (n = 81) were Gal3ST1 positive; the values for pRCC and chRCC were 76% (n = 13) and 25% (n = 2), respectively (Table 2). Taken together, Gal3ST1 expression was significantly less associated to chRCC than to other 2 histological types (p = 0.001; Table 2). Strong expression of Gal3ST1 (IG = 3) was detected in 36% (n = 34) ccRCC, 18% (n = 3) pRCC and 12,5% (n = 1) chRCC (Table 3). There was no association of Gal3ST1 expression with patient age; interestingly, strong Gal3ST1 expression was significantly more frequent in male than in female patients (p = 0.04; Table 2), also when accounting for histology (p = 0.04). Nevertheless, in the TCGA dataset (described below) we could not observe a significant association between Gal3ST1 mRNA expression and gender.

**Table 2:** Association of intensity of Gal3ST1 expression with risk factors (Fisher’s exact test).

Parameter	Level	IG 0	IG 0.5	IG 1	IG 2	IG 3	p
N		23	20	11	27	38	
Sex (%)	m	16 (69.6)	12 (60.0)	5 (45.5)	19 (70.4)	33 (86.8)	0.043
	w	7 (30.4)	8 (40.0)	6 (54.5)	8 (29.6)	5 (13.2)	
Age (median [IQR])		63.00 [52.00–71.50]	68.00 [61.50–71.50]	69.00 [54.00- 72.00]	62.00 [54.00–66.50]	64.00 [53.50- 70.00]	0.539
T stage (%)	pT1	12 (52.2)	11 (55.0)	5 (45.5)	14 (51.9)	21 (55.3)	0.648
	pT2	6 (26.1)	3 (15.0)	3 (27.3)	3 (11.1)	3 (7.9)	
	pT3/4	5 (21.7)	6 (30.0)	3 (27.3)	10 (37.0)	14 (36.8)	
Grade (%)	G1	3 (17.6)	2 (10.5)	2 (18.2)	3 (11.1)	4 (10.8)	0.862
	G2	11 (64.7)	15 (78.9)	8 (72.7)	23 (85.2)	29 (78.4)	
	G3	3 (17.6)	2 (10.5)	1 (9.1)	1 (3.7)	4 (10.8)	
N Status (%)	0	22 (95.7)	18 (90.0)	11 (100.0)	25 (92.6)	38 (100.0)	0.243
	1	1 (4.3)	2 (10.0)	0 (0.0)	2 (7.4)	0 (0.0)	
M Status (%)	M0	20 (87.0)	20 (100.0)	9 (81.8)	23 (85.2)	35 (92.1)	0.302
	M1	3 (13.0)	0 (0.0)	2 (18.2)	4 (14.8)	3 (7.9)	
Histology (%)	ccRCC	13 (56.5)	15 (75.0)	9 (81.8)	23 (85.2)	34 (89.5)	0.026
	pRCC	4 (17.4)	4 (20.0)	2 (18.2)	4 (14.8)	3 (7.9)	
	chRCC	6 (26.1)	1 (5.0)	0 (0.0)	0 (0.0)	1 (2.6)	
chRCC (%)	no	17 (73.9)	19 (95.0)	11 (100.0)	27 (100.0)	37 (97.4)	0.004
	yes	6 (26.1)	1 (5.0)	0 (0.0)	0 (0.0)	1 (2.6)	
V Status (%)	0	18 (78.3)	15 (75.0)	8 (72.7)	22 (81.5)	31 (81.6)	0.703
	1	3 (13.0)	5 (25.0)	3 (27.3)	3 (11.1)	4 (10.5)	
	2	2 (8.7)	0 (0.0)	0 (0.0)	2 (7.4)	3 (7.9)	
RSF (%)	0	21 (91.3)	20 (100.0)	11 (100.0)	25 (92.6)	36 (94.7)	0.799
	1	2 (8.7)	0 (0.0)	0 (0.0)	2 (7.4)	2 (5.3)	
R Status (%)	0	21 (95.5)	19 (95.0)	11 (100.0)	24 (96.0)	36 (94.7)	1.000
	1/2	1 (4.5)	1 (5.0)	0 (0.0)	1 (4.0)	2 (5.3)	
L Status (%)	0	23 (100.0)	18 (90.0)	11 (100.0)	26 (96.3)	37 (97.4)	0.545
	1	0 (0.0)	2 (10.0)	0 (0.0)	1 (3.7)	1 (2.6)	

*IG* intensity grade, *RSF* renal sinus fat infiltration.

**Table 3:** Association of Gal3ST1 expression (negative vs. positive) with risk factors (Fisher’s exact test).

parameter	level	Gal3ST—(%)	Gal3ST + (%)	p
n		23	96	
Sex (%)	m	16 (69.6)	69 (71.9)	0.802
	w	7 (30.4)	27 (28.1)	
Age (median [IQR])		63.00 [52.00—71.50]	64.00 [55.00–70.00]	0.699
T Stage (%)	pT1	12 (52.2)	51 (53.1)	0.217
	pT2	6 (26.1)	12 (12.5)	
	pT3/4	5 (21.7)	33 (34.4)	
Grading (%)	G1	3 (17.6)	11 (11.7)	0.300
	G2	11 (64.7)	75 (79.8)	
	G3	3 (17.6)	8 (8.5)	
N Status (%)	0	22 (95.7)	92 (95.8)	1.000
	1	1 (4.3)	4 (4.2)	
M Status (%)	M0	20 (87.0)	87 (90.6)	0.699
	M1	3 (13.0)	9 (9.4)	
Histology (%)	ccRCC	13 (56.5)	81 (84.4)	< 0.001
	pRCC	4 (17.4)	13 (13.5)	
	chRCC	6 (26.1)	2 (2.1)	
chRCC (%)	no	17 (73.9)	94 (97.9)	0.001
	yes	6 (26.1)	2 (2.1)	
V Status (%)	0	18 (78.3)	76 (79.2)	0.817
	1	3 (13.0)	15 (15.6)	
	2	2 (8.7)	5 (5.2)	
RSF* (%)	0	21 (91.3)	92 (95.8)	0.328
	1	2 (8.7)	4 (4.2)	
Sarcomatoid morphology (%)	0	23 (100.0)	95 (99.0)	1.000
	1	0 (0.0)	1 (1.0)	
R Status (%)	0	21 (95.5)	90 (95.7)	1.000
	1/2	1 (4.5)	4 (4.3)	
L Status (%)	0	23 (100.0)	92 (95.8)	1.000
	1	0 (0.0)	4 (4.2)	

*RSF renal sinus fat infiltration.

### Gal3ST1 protein expression in RCC does not correlate with established pathological risk factors and is not associated with progression-free survival

In order to investigate the association of Gal3ST1 positivity with tumor progression, we first checked the representability of our study cohort in regard to the known standard risk factors (pT- and N-stage; L-, N- and V-status; R-Status; histological grade; sarcomatoid differentiation and infiltration of renal sinus fat tissue). Prognostic value of these conventional risk categories could be confirmed in our cohort with highly significant p-values (Table 4 & suppl. Figure 1). Regarding the histological type of RCC, ccRCC showed a tendency towards worse outcome in comparison to other two entities, albeit here without statistical significance (p = 0.406; suppl. Figure 1f). The detailed relation of established prognostic factors to (PFS) probability in our cohort is shown in suppl. Figure 1.

**Table 4:** Univariable Cox regression on progression-free survival.

parameter	N	Events	level	ref	HR	LCL	UCL	Waldp	LRTp
Gal3 (binary)	119	29	pos	neg	1,7	0,59	4,89	0.327	0.297
Gal3 (IG)*	119	29	0.5	0	1,97	0,55	7	0.297	0.373
			1	0	2,58	0,57	11,66	0.219	
			2	0	2,35	0,72	7,66	0.158	
			3	0	1,07	0,31	3,64	0.920	
Grade	111	28	G2	G1	3,97	0,54	29,37	0.177	0.031
			G3	G1	12,86	1,42	116,38	0.023	
T Stage	119	29	pT2	pT1	2,96	0,94	9,35	0.064	< 0.001
			pT3/4	pT1	7,39	3,02	18,09	< 0.001	
Histology	119	29	pRCC	ccRCC	0,45	0,11	1,9	0.278	0.347
			chRCC	ccRCC	0,45	0,06	3,3	0.429	
V status	119	29	1	0	6,77	3,03	15,12	< 0.001	< 0.001
			2	0	5,6	1,59	19,73	0.007	
N status	119	29	1	0	9,32	3,45	25,16	< 0.001	< 0.001
M status	119	29	M1	M0	21,4	8,01	57,15	< 0.001	< 0.001
RSF*	119	29	1	0	4,87	1,66	14,32	0.004	0.016
R status	116	28	1/2	0	5,63	1,92	16,48	0.002	0.009
L status	119	29	1	0	9,83	3,24	29,83	< 0.001	0.001

*IG intensity grade, *RSF* renal sinus fat infiltration, *HR* hazard ratio, *LCL/UCL* lower/upper 95% confidence limits, *Waldp* p-value of Wald test, *LRTp* p-value of likelihood-ratio test.

**Figure 2:**
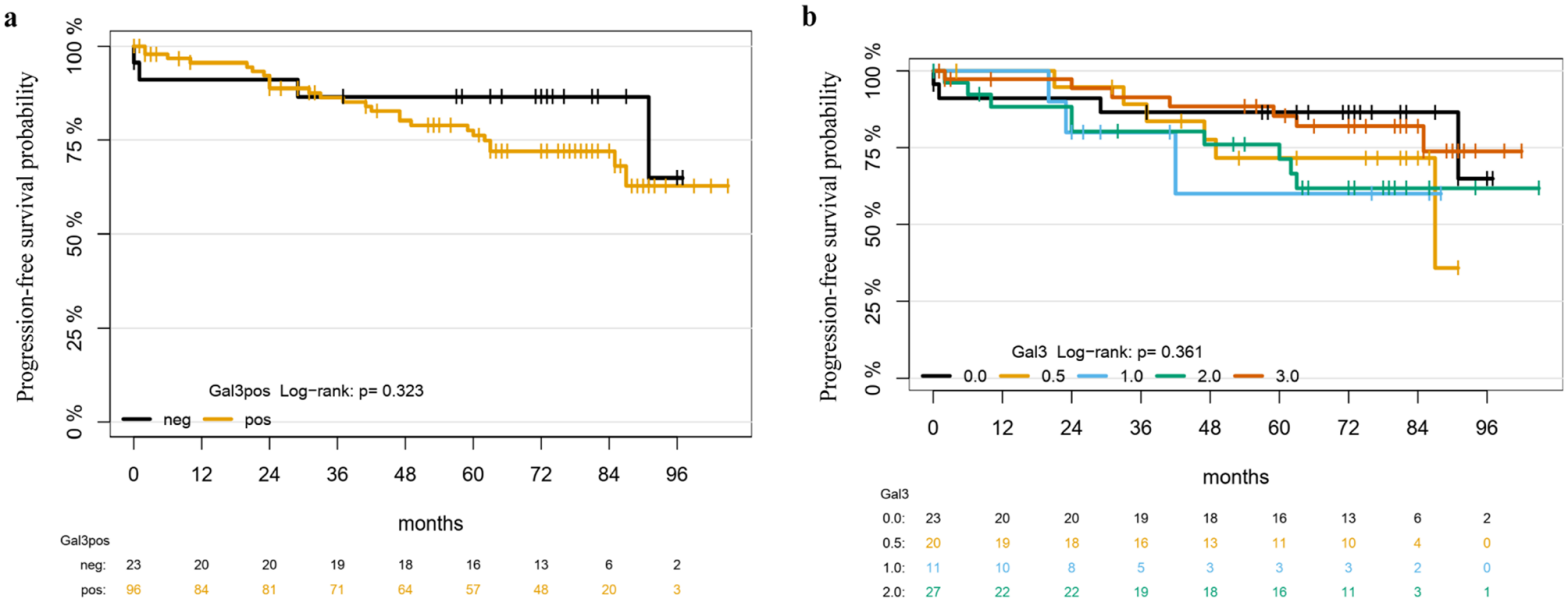
Kaplan–Meier curves for progression-free survival according to (**a**) binary expression of Gal3ST1 (positive vs negative) and (**b**) intensity grades of Gal3ST1 protein expression in our patient cohort.

To investigate the prognostic relevance of Gal3ST1 expression in RCC, we analyzed the association of Gal3ST1 positivity to single classical risk factors and performed univariable Cox-Regression on PFS. We employed 2 comparison modules: (1) binary module (influence of Gal3ST1 positivity in general); and (2) quantitative module (impact of Gal3ST1 appreciating the intensity of the enzyme expression). In both cases no significant association with known risk factors could be detected (Tables 2 and 3). In addition, due to a different biology and behavior of pRCC types 1 and 2, we performed separate analysis of the two entities in regard to classical prognostic factors and Gal3ST1 expression. Our results go in line with already described initial presentation of pRCC type 2 at higher stage (p = 0.02 for pT-status) and show no significant differences in the Gal3ST1 positivity (p = 1.00 for the binary model and p = 0.83 for quantitative model; Table 5). Finally, Gal3ST1 expression in our cohort had no significant influence on PFS (Fig. 2; readout parameters: cancer recurrence and late metastasis—in total 29 events, HR 1.7 95%CI (0.6–4.9)). The detailed data on impact of Gal3ST1 on PFS are presented in the Table 4. When additionally accounting for classical risk factors in multivariable Cox-model, the effect observed in univariable Cox-regression further diminished (HR: 1.03 and 1.35; Tables 6 and 7, respectively).

**Table 5:** Association of pRCC histotypes 1 and 2 with classical risk factors and Gal3ST1 expression (Fisher’s exact test).

Parameter	Level	pRCC type 1	pRCC type 2	p
n		10	7	
Sex (%)	m	8 (80.0)	5 (71.4)	1.000
	w	2 (20.0)	2 (28.6)	
Age (median [IQR])		58.50 [50.75, 66.50]	73.00 [66.50, 73.50]	0.157
T Stage (%)	pT1	9 (90.0)	3 (42.9)	0.026
	pT2	1 (10.0)	1 (14.3)	
	pT3/4	0 (0.0)	3 (42.9)	
Grading (%)	G1	3 (30.0)	1 (14.3)	0.369
	G2	7 (70.0)	4 (57.1)	
	G3	0 (0.0)	2 (28.6)	
N Status (%)	0	10 (100.0)	6 (85.7)	0.412
	1	0 (0.0)	1 (14.3)	
M Status (%)	M0	10 (100.0)	4 (57.1)	0.051
	M1	0 (0.0)	3 (42.9)	
V Status (%)	0	10 (100.0)	5 (71.4)	0.154
	1	0 (0.0)	1 (14.3)	
Gal3ST1 (Int*)	0/0.5	5 (50.0)	3 (42.9)	0.665
	1/2	4 (40.0)	2 (28.6)	
	3	1 (10.0)	2 (28.6)	
Sarcomatoid morphology (%)	0	10 (100.0)	7 (100.0)	1.000
	1	0 (0.0)	0 (0.0)	
R Status (%)	0	10 (100.0)	6 (100.0)	1.000
	1/2	0 (0.0)	0 (0.0)	
L Status (%)	0	10 (100.0)	6 (85.7)	0.412
	1	0 (0.0)	1 (14.3)	

*Int Expression intensity.

**Table 6:** Multivariable Cox regression model (n = 119, number of events = 29).

Variable	Units	HazardRatio	CI.95	p-value
Gal3pos	neg	Ref		
	pos	1.03	[0.33;3.25]	0.95624
Tumor stage	pT1	Ref		
	pT2	1.60	[0.45;5.65]	0.46356
	pT3/4	3.89	[1.38;11.00]	0.01028
N status	0	Ref		
	1/2	4.61	[1.47;14.50]	0.00887
M status	M0	Ref		
	M1	16.13	[5.30;49.11]	< 0.001
Histology	ccRCC	Ref		
	pRCC	0.44	[0.09;2.22]	0.31959
	chRCC	0.72	[0.09;5.69]	0.75179

**Table 7:** Multivariable Cox regression model (n = 111, number of events = 28).

Variable	Units	Missing	HazardRatio	CI.95	p-value
Gal3pos	neg	0	Ref		
	pos		1.35	[0.37;4.85]	0.6504
TumorStage	pT1	0	Ref		
	pT2		1.74	[0.50;6.12]	0.3845
	pT3/4		3.76	[1.34;10.57]	0.0121
N.Status	0	0	Ref		
	1/2		3.90	[1.23;12.35]	0.0209
M.Status	M0	0	Ref		
	M1		15.83	[5.02;49.92]	< 0.001
Grading	G1	8	Ref		
	G2		1.93	[0.25;15.09]	0.5325
	G3		2.37	[0.22;25.96]	0.4806

### mRNA expression of Gal3ST1 mRNA in TCGA cohort

**Figure 3:**
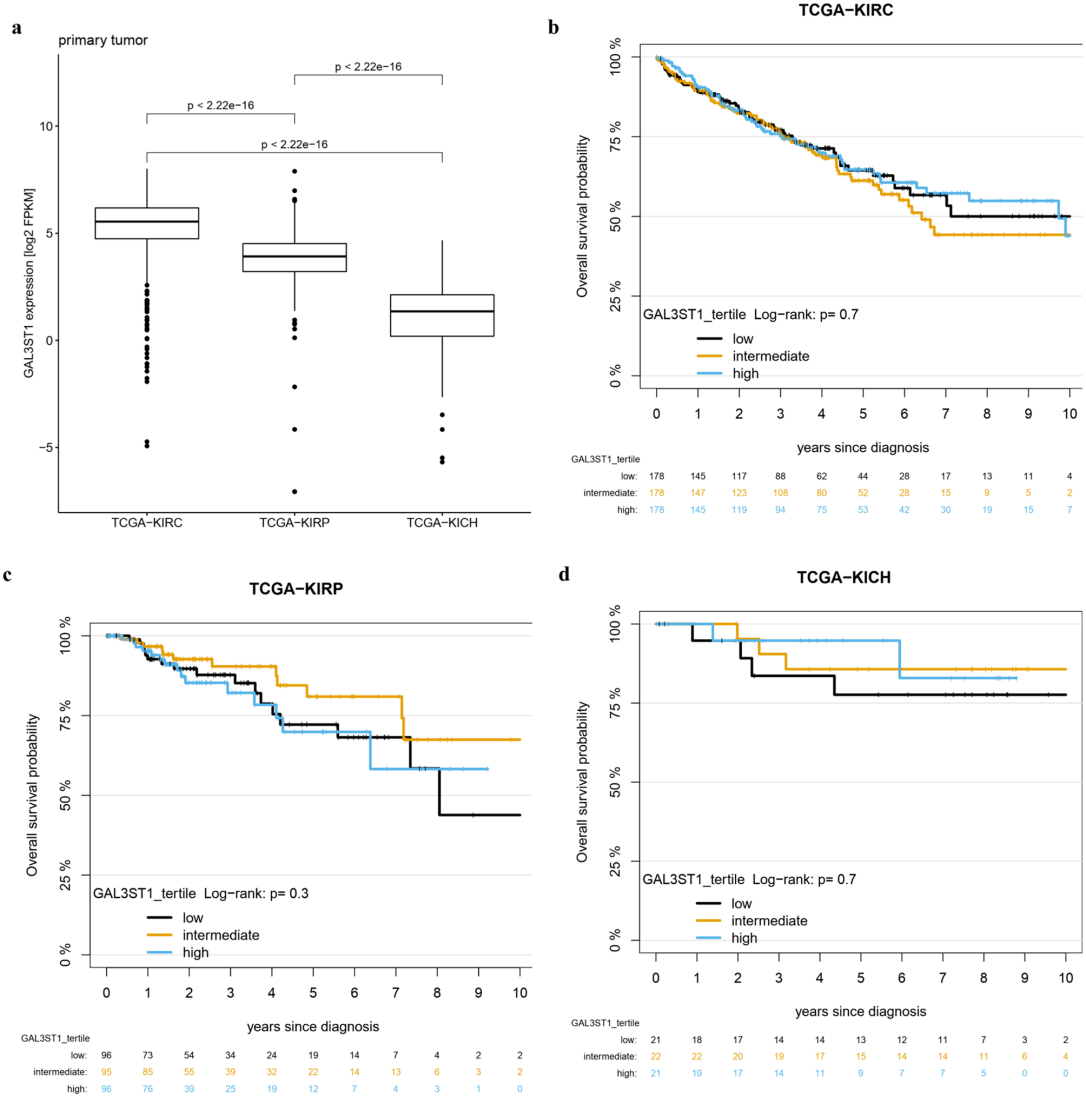
(**a**) Analysis of Gal3ST1 mRNA expression in TCGA cohort of ccRCC (‘KIRC’), pRCC (‘KIRP’) and chRCC (‘KICH’) patients. (**b**) Kaplan–Meier curves for overall survival probability for three histotypes from TCGA using tertile cut-offs for Gal3ST1 mRNA expression.

In the TCGA dataset including ccRCC (n = 534), pRCC (n = 288) and chRCC (n = 65), Gal3ST1 showed differential mRNA expression between the selected histological entities, with strongest expression in ccRCC and lowest in chRCC (p < 2.22e−16 between each RCC histotype, Fig. 3a). Subtype information of pRCC was available for only a subset of cases (Suppl. Figure 2). Similar to our cohort, no difference in expression was observed between pRCC type 1 (n = 74) and type 2 (n = 60; p = 0.7).

Interestingly, ccRCC and pRCC in the TCGA cohort showed significantly higher Gal3ST1 mRNA expression than non-neoplastic kidney tissue of the same patients, opposite to the chRCC (data not shown); still, without knowing the exact localization of the ‘normal kidney tissue’ (cortex vs. medulla), this observation should be interpreted with caution. Importantly, regarding the influence of the Gal3ST1 mRNA expression on overall survival, in the TCGA cohort we could not detect a significant effect in any of the RCC categories (p = 0.3–0.7; Fig. 3b–d), going in line with the protein expression data from our patient cohort.

## Discussion

Despite a large number of investigated histological and clinical RCC features as hallmarks of malignancy, only a few have been to date confirmed and accepted as independent prognostic parameters^37–39^. The role of specific parameters of RCC microenvironment in immune escape and cancer progression remains poorly described. Sulfatides are synthesized by renal tumors and can serve as tumor antigens for unconventional T cells^15–20^. Appreciating a line of data on abundancy of sulfated glycosphingolipids in renal tumors and their predictive value regarding lymph node metastases in colon cancer patients^23,28^, we have previously addressed the role of sulfatide on modification of renal cell cancer-associated macrophage phenotype in a murine ex vivo / in vitro model and have shown its potential immunomodulatory effect^29^. Hence, sulfatide excess in RCC-micromilieu potentially contributes to immune escape of cancer cells and tumor progression. As lipids in general can be immunohistochemically detected only in frozen tissue samples, a question of applicability of protein components of sulfatide pathway on formalin-fixed, paraffin-embedded tissue as direct risk factors of RCC progression has been raised. Especially the first downstream component of the pathway—sulfatide-producing enzyme, Gal3ST1—has been extensively investigated in past years. Using distinct kidney tumor cell lines, recently published data convincingly show enhanced binding of Gal3ST1-expressing renal cancer cells to platelets that resulted in protection of neoplastic cells against natural killer cell-triggered cytotoxic response, thus suggesting the tumor immune escape and worse prognosis^35^. Moreover, the authors identify HIF-mediated increased synthesis of Gal3ST1, implying the effect of hypoxic tumor microenvironment on Gal3ST1 expression. In line with these observations, pathology atlas of human cancer transcriptome reports Gal3ST1 as one of the most upregulated genes in ccRCC samples in comparison to normal kidney tissue^40^. In an animal model employing nude mice, others report increased intrahepatic metastasis of hepatocellular carcinoma cells upon transfection of Gal3ST1^41^. Considering the observations on physiological protective role of renal sulfatide in metabolic acidosis^21^, it is challenging to hypothesize that, in pathologic setting of neoplastic disease, increased sulfatide content represents adaptive response to typically acidic and hypoxic tumor micromilieu and indirectly influences immune landscape of the TME^42^. Still, the prognostic significance of direct Gal3ST1 detection in clinical RCC setting has been to date not confirmed.

We evaluated here the prognostic value of Gal3ST1 protein expression in human RCC tissue. In our patient cohort, we could not detect association of immunohistochemical Gal3ST1 expression with progression-free survival (including recurrence- and late metastasis-free survival). Also, our study showed no significant association of Gal3ST1 protein expression (appreciating different expression intensities as well) with to date well defined prognostic parameters of RCC. Despite two major statistical limitations of our cohort—relatively small sample size and heterogeneity, (1) classical risk factors showed here significant impact on the outcome and thereby confirmed the representative character of the cohort, (2) multivariate confounder-adjusted analysis showed similar results and (3) supporting our data, the analysis of mRNA expression of Gal3ST1 from an independent, larger representative TCGA dataset of RCC revealed no significant impact of Gal3ST1 on overall survival. Nevertheless, it cannot be excluded that in larger cohort with more events—metastases and/or recurrent disease cases—GalsST1 might also reach statistical significance. In addition to the prognostic significance, lower Gal3ST1 expression in chRCC in comparison to ccRCC and pRCC in our patient cohort but also in TCGA dataset suggests that this marker might help to differentiate between these entities. However, further investigation addressing diagnostic significance of Gal3ST1 in distinct histological RCC types is needed to strengthen this observation.

Finally, our results indicate that, in major histological RCC types that make > 95% of RCC cases worldwide, Gal3ST1 does not qualify as an independent prognostic marker. The possible discrepancy between previous reports (from cell culture and murine models) and results of our clinical retrospective follow-up study underscores the need to better understand the (substrate) GalCer—(enzyme) Gal3ST1—(product) sulfatide axis in the RCC microenvironment.

## Material and methods

### Patients, sampling criteria and tissue processing

Patients included in the cohort underwent radical or partial nephrectomy in the period between February 2008 and July 2011 at the Department of Urology and Urosurgery of the Medical Centre Mannheim, University of Heidelberg. All of the patients consented to the use of their tissue in this study and the procedure was approved by the Ethic Committee of the University Medical Centre Mannheim, University of Heidelberg (Reference number: 2015-549N-MA). Clinical data were collected by chart review. Therapy, disease progression and survival follow-up were obtained by contacting the urologist and general practitioners of the respective patients. The patients received similar therapy, according to guidelines at the time of clinical presentation (TKI mono-therapy in first line). The tissue samples were routinely fixed in 4% paraformaldehyde (PFA) and consecutively embedded in paraffin to obtain the paraffin sections for standard tissue staining (hematoxylin–eosin—HE; periodic acid Schiff—PAS) and immunohistochemistry. The histological diagnosis was performed by experienced pathologists at the Institute of Pathology, Medical Centre Mannheim, University of Heidelberg according to the actual WHO classification of Tumours of the Urinary tract. For diagnostic purposes (2008–2011), cancer staging and grading was originally performed based on the criteria of the TNM Classification of Malignant Tumors at the time of diagnosis^43^. Tumor stage and grade was newly re-evaluated for the purpose of this study, according to the actual 8th edition of the TNM Classification (Union for International Cancer Control—UICC). Of note, in our patient cohort, no discrepancies in TNM-stage and grade could be detected when comparing criteria from different TNM editions, as approved independently by two pathologists (ZVP and SP). To avoid an overlap of formerly defined histologic RCC types with meanwhile newly introduced entities (WHO Classification of Tumours of the Urinary System and Male Genital Organs, 3rd vs. 4th Edition), only the patients with clear-cut criteria for ccRCC, pRCC and chRCC were involved in the study. Due to the absence of clearly defined diagnostic algorithm and/or to low number of patients, 3 patients with diagnosis of ‘RCC, unclassified’ and a single patient with collecting duct (ductus Bellini) carcinoma were excluded from the study. Finally, our cohort included 119 patients.

### Immunohistochemistry and sample evaluation

For immunohistochemical Gal3ST1 staining, paraffin-embedded tissue was cut to 1-µm sections. Polyclonal rabbit anti-human Gal3ST1-antibody was applied at final concentration of 2 µg/ml. Image acquisition and analysis of immunohistochemical and H&E stained slides was done using the PreciPoint scanning microscope M8 with Olympus PlanCN 20x/0.65 Objective and MicroPoint software (v.2016-02-05; PreciPoint, Freising, Germany). Necrotic, fibrotic or hemorrhagic tumor areas were not assessed. The intensity of Gal3ST1 staining in vital tumor was evaluated by two pathologists (ZVP and SP) and scored using a semiquantitative scoring system: 0- no specific staining of tumor cells; 0,5- focal single cell staining; 1- weak patchy staining; 2- moderate staining; and 3- strong diffuse positivity. Internal positive control was physiological cytoplasmic positivity of proximal and distal tubuli.

### TCGA analysis

RNA-Seq expression data of GAL3ST1 in primary tumor samples for the cohorts ‘TCGA-KIRC’ (ccRCC, n = 534), ‘TCGA-KIRP’ (pRCC, n = 288) and ‘TCGA-KICH’ (chRCC, n = 65) were downloaded from the GDC data portal using the Bioconductor package TCGAbiolinks. GAL3ST1 expression was analyzed as log2-transformed FPKM (Fragments Per Kilobase Million) values.

Overall survival (OS) was analyzed as time from diagnosis. GAL3ST1 expression was analyzed for association with OS based on Kaplan–Meier estimates and log-rank test in each cohort separately using tertile expression cut-offs.

### Statistics

Fisher’s exact test and Wilcoxon test were used to assess the association between GaL3ST1 expression and clinic-pathological factors. Primary endpoint was progression-free survival (PFS) and included local recurrence and new metastases. PFS was estimated by the Kaplan–Meier method. Log-rank test and Cox regression models were used to assess the prognostic impact of GaL3ST1 and established risk factors. Analysis was performed with statistical software R.

### Informed consent

Informed consent was obtained from all participants in this study and the procedure was approved by the Ethic Committee of the University Medical Centre Mannheim, University of Heidelberg (Reference number: 2015-549N-MA). All research was performed in accordance with relevant guidelines and regulations.

## Supplementary Information

Supplementary legend.

Supplementary figure 1.

Supplementary figure 2.
