# Supplementary material for: The prognostic value of galactosylceramide-sulfotransferase (Gal3ST1) in human renal cell carcinoma
Source: Sci Rep. 2021 May 25;11:10926. doi: 10.1038/s41598-021-90381-6 (PMC8149814; doi:10.1038/s41598-021-90381-6)
Supplement: Supplementary file 1 — Supplementary legend. [file 41598_2021_90381_MOESM1_ESM.docx]

**The prognostic value of galactosylceramide-sulfotransferase (Gal3ST1) in human renal cell carcinoma**

Stefan Porubsky^1,6^, Malin Nientiedt^2^, Maximilian C. Kriegmair^2^, Jörn-Helge Heinrich Siemoneit^1^, Roger Sandhoff^3^, Richard Jennemann^3^, Hendrik Borgmann^4^,Timo Gaiser^1^, Cleo-Aron Weis^1^, Philipp Erben^2^, Thomas Hielscher^5^ and Zoran V. Popovic^1*^

*Corresponding address:

Zoran.Popovic@umm.de

**Supplementary information**

**Supplementary figure 1.** Kaplan-Meier curves for progression-free survival related to standard prognostic risk factors: a) grade (based on ISUP/WHO criteria), b) pT stage, c) V status, d) M status, e) R status and f) histological type.

**Supplementary figure 2.** Boxplots of Gal3ST1 mRNA expression in pRCC type 1 and pRCC type 2 cases from the TCGA dataset.
