# Supplementary figures and images for: The prognostic value of galactosylceramide-sulfotransferase (Gal3ST1) in human renal cell carcinoma
Source: Sci Rep. 2021 May 25;11:10926. doi: 10.1038/s41598-021-90381-6 (PMC8149814; doi:10.1038/s41598-021-90381-6)

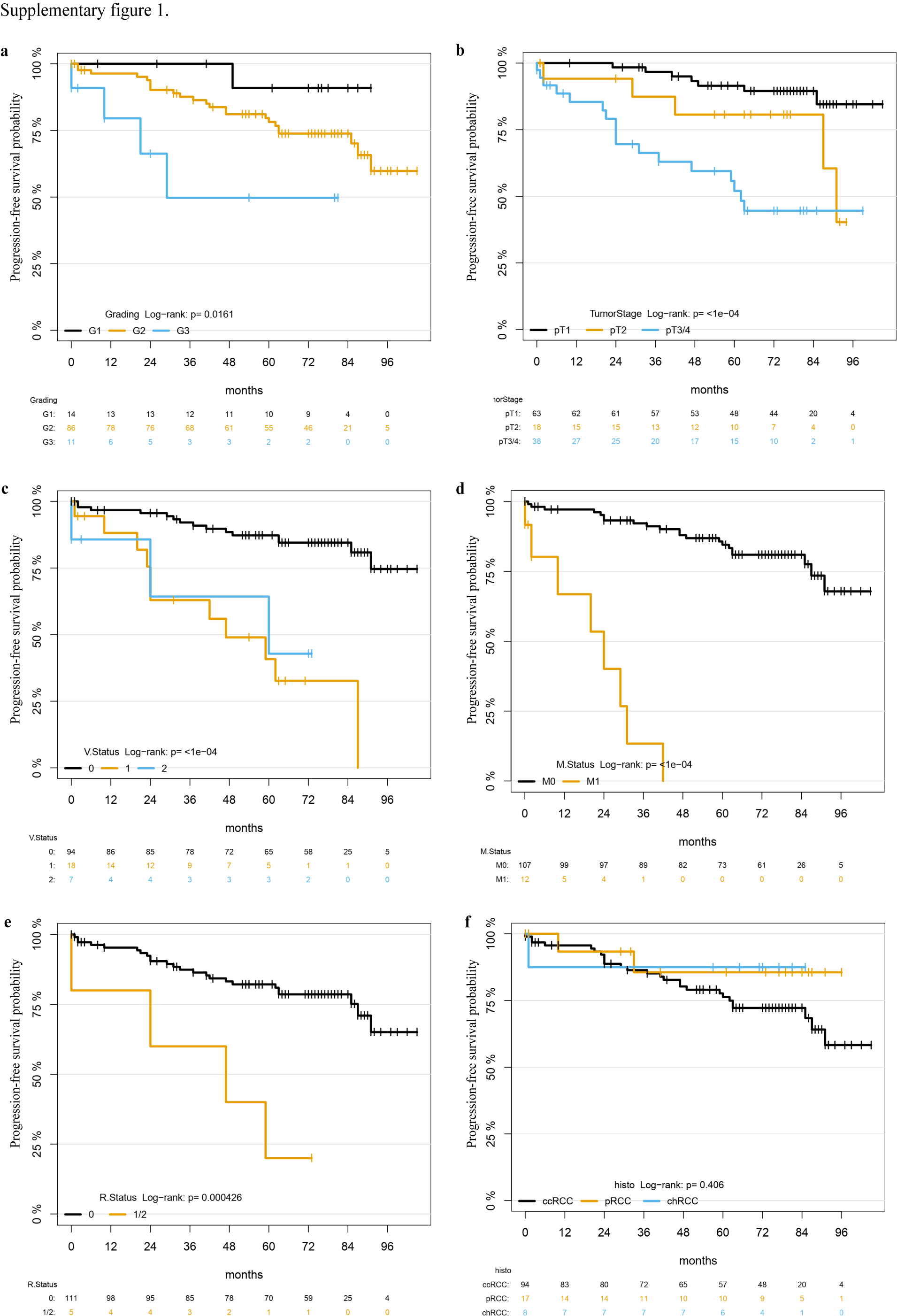

Supplement: Supplementary file 2 — Supplementary figure 1. [file 41598_2021_90381_MOESM2_ESM.tif]

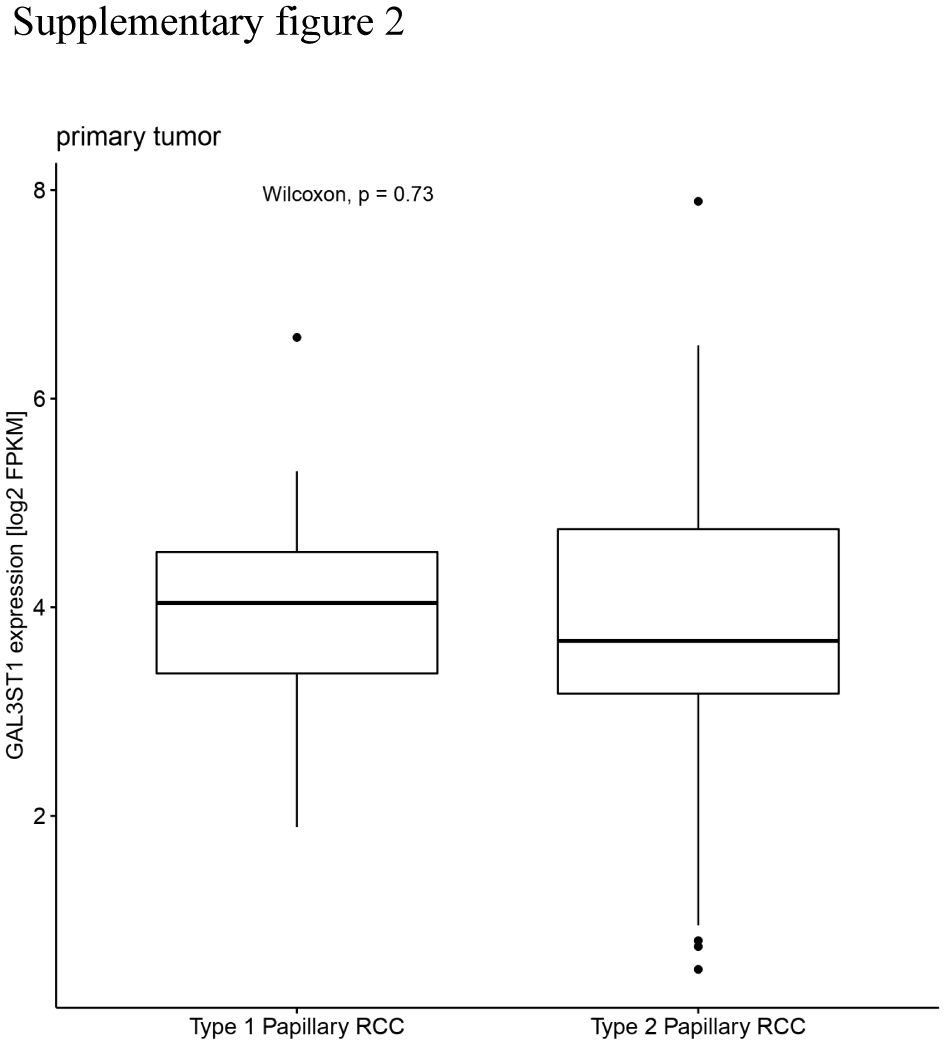

Supplement: Supplementary file 3 — Supplementary figure 2. [file 41598_2021_90381_MOESM3_ESM.tif]
